# Supplementary material for: Phosphoproteomic screening identifies Rab GTPases as novel downstream targets of PINK1
Source: EMBO J. 2015 Oct 16;34(22):2840–61. doi: 10.15252/embj.201591593 (PMC4654935; doi:10.15252/embj.201591593)
Supplement: Supplementary file 1 — Appendix [file EMBJ-34-2840-s001.pdf]

Phosphoproteomic screening identifies Rab GTPases as novel downstream targets of PINK1

Yu-Chiang Lai, Chandana Kondapalli, Ronny Lehneck, James B. Procter, Brian D. Dill, Helen I. Woodroof, Robert Gourlay, Mark Peggie, Thomas J. Macartney[4], Olga Corti, Jean-Christophe Corvol, David G. Campbell, Aymelt Itzen, Matthias Trost, and Miratul M. K. Muqit

Appendix

Table of contents

Appendix Figure S1-S12

Appendix Fig S1

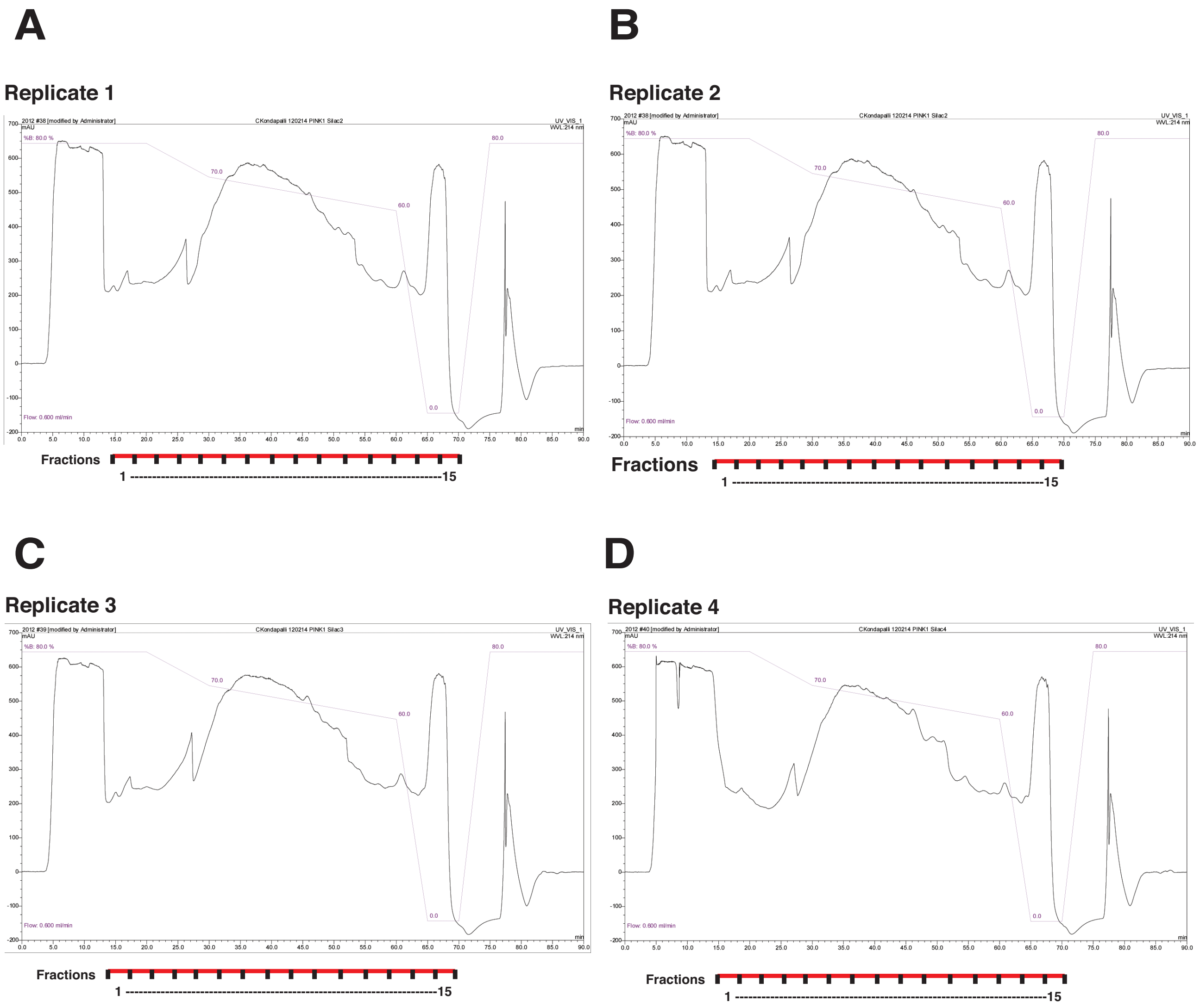

Appendix Fig S1. HILIC (Hydrophilic Interaction Liquid Chromatography) chromatogram. A-D: HILIC Chromatograms for all four experimental replicates employed. The chromatogram represents absorbance of the peptides eluted (in mAU) on the y-axis and retention time on the x-axis. Fractions enriched with phospho-peptides (1-15) were collected for TiO_2 enrichment.

Appendix Fig S2

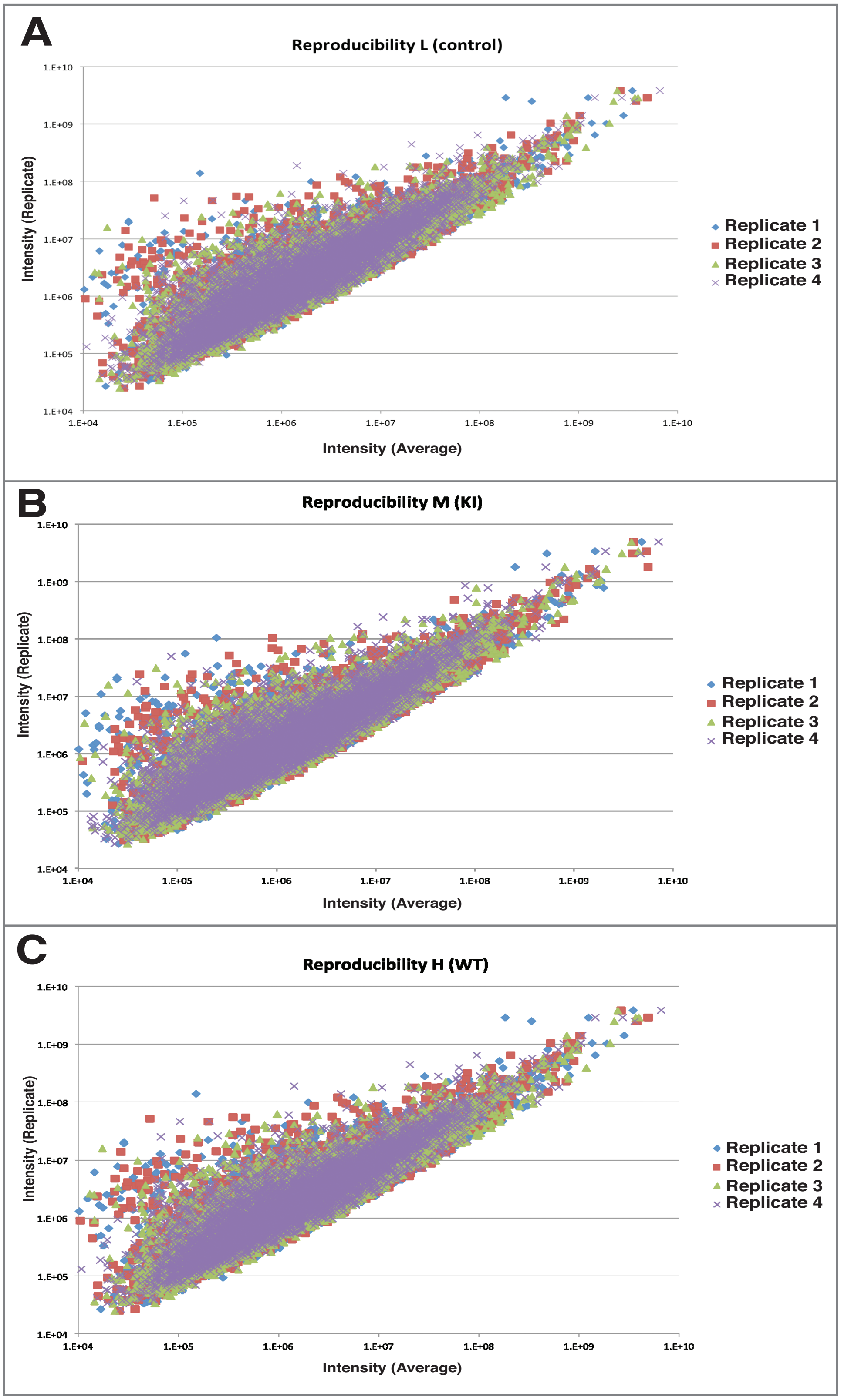

Appendix Fig S2. Inter-experimental reproducibility of proteomic data. Scatter plots showing a comparison of peptide intensity from each independent experimental replicate in (A) unlabeled condition, (B) 'medium' labeled condition and (C) 'heavy' labeled condition, with a strong correlation with the average peptide intensity.

Appendix Fig S3

A

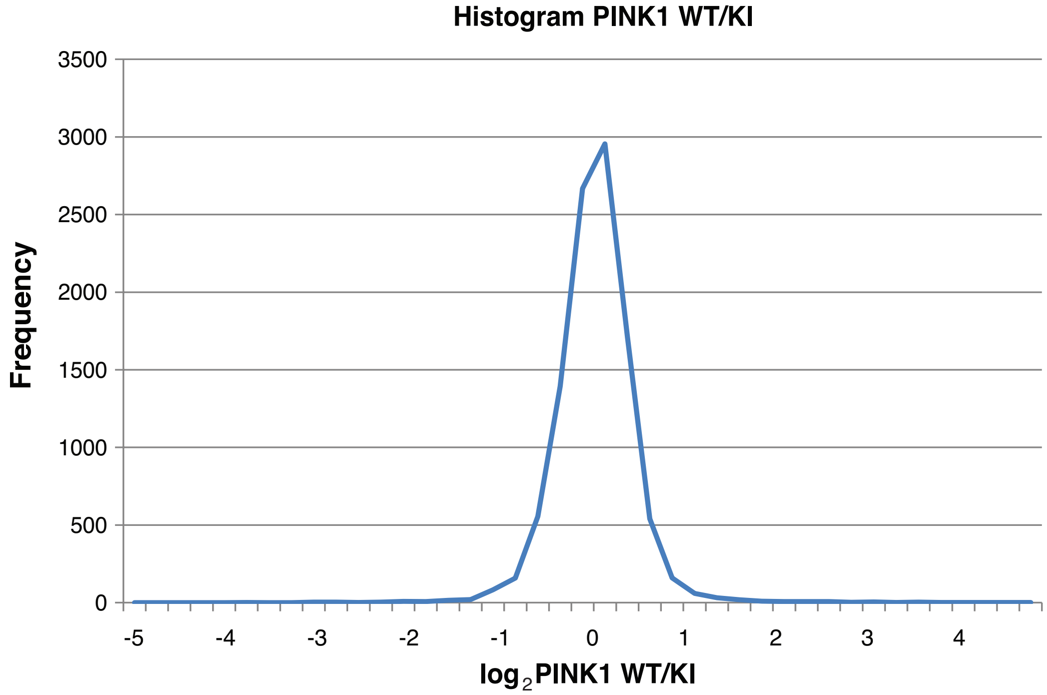

B

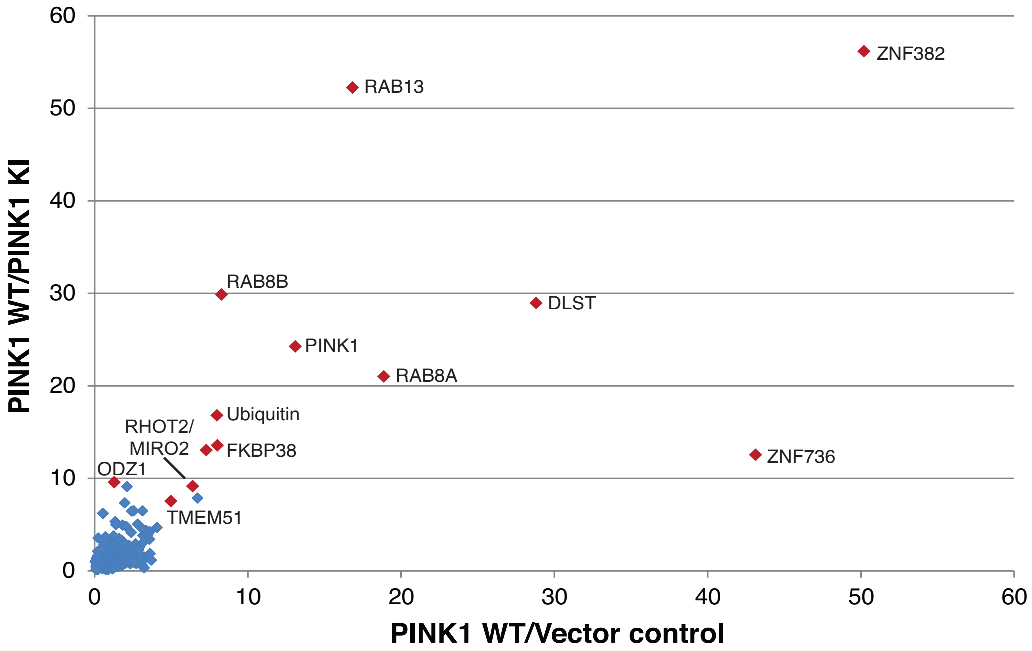

Appendix Fig S3. Analysis of PINK1-regulated phosphoproteome A: Distribution of phosphopeptides identified frequency for WT PINK1/ KI PINK1. Note that majority of the phosphopeptides remain unchanged between the 'median' (KI PINK1) and 'heavy' (WT PINK1) populations and hence have a value close to 0 on a Log_2 X-axis. **B:** Comparison of average ratio of phosphopeptide between WT PINK1/KI PINK1 and WT PINK/empty vector.

Appendix Fig S4

A

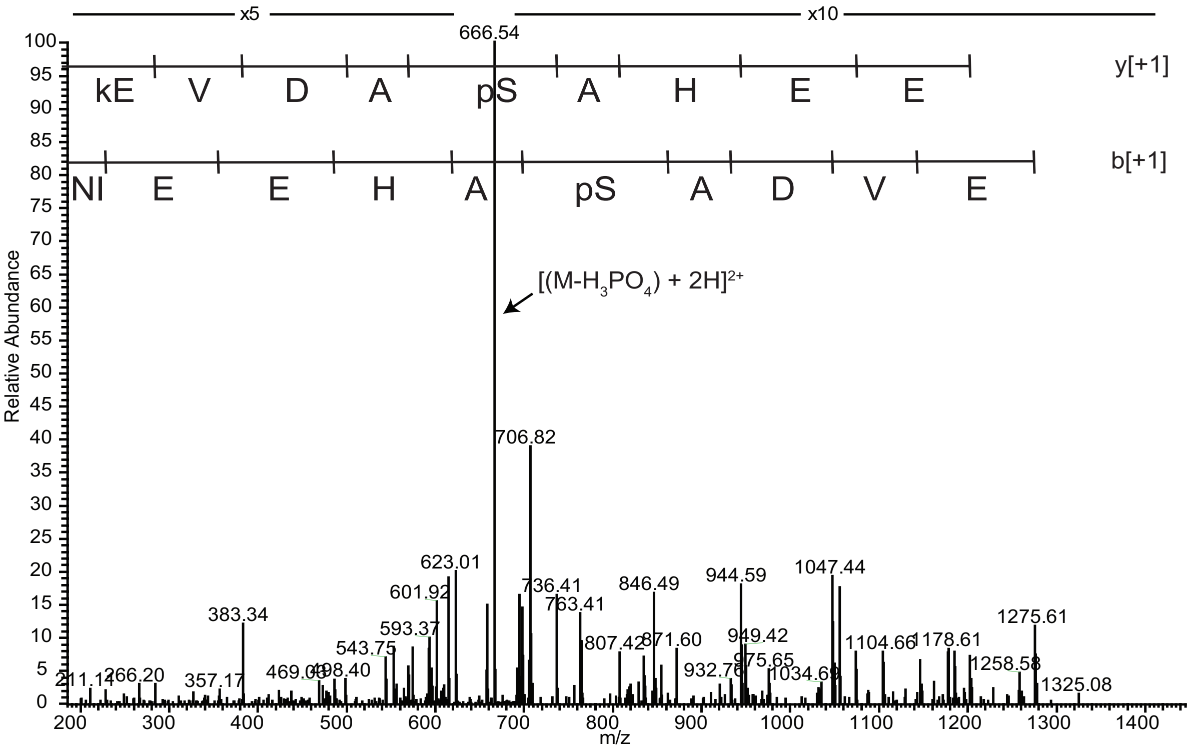

B

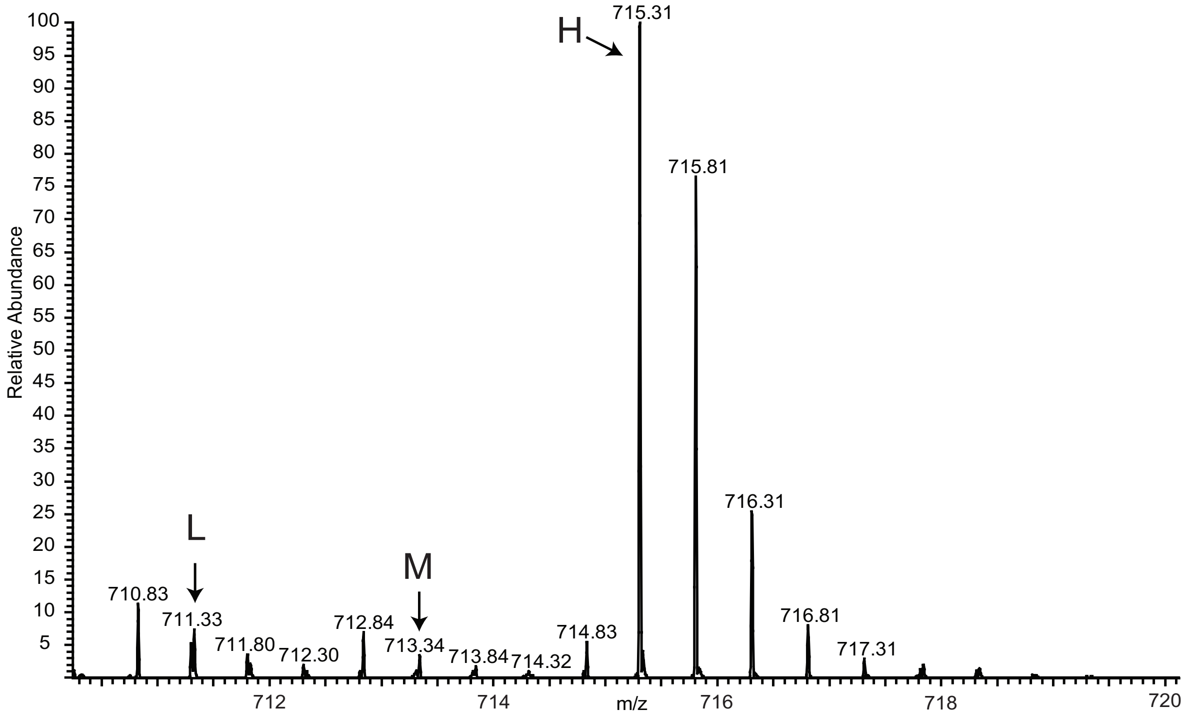

Appendix Fig S4. MS/MS fragmentation spectra of the phosphorylated Rab8A peptide NIEEHApSADVEK. A: MS/MS spectrum of the "heavy" phospho-peptide NIEEHApSADVEk (where k is the K8 SILAC amino acid) of Rab8A. **B:** The 'heavy' (H), 'medium' (M) and 'light' (L) peptides that differ by 2 m/z are identified in cells stably expressing wild-type PINK1-FLAG, kinase inactive PINK1-FLAG or FLAG empty, respectively.

Appendix Fig S5

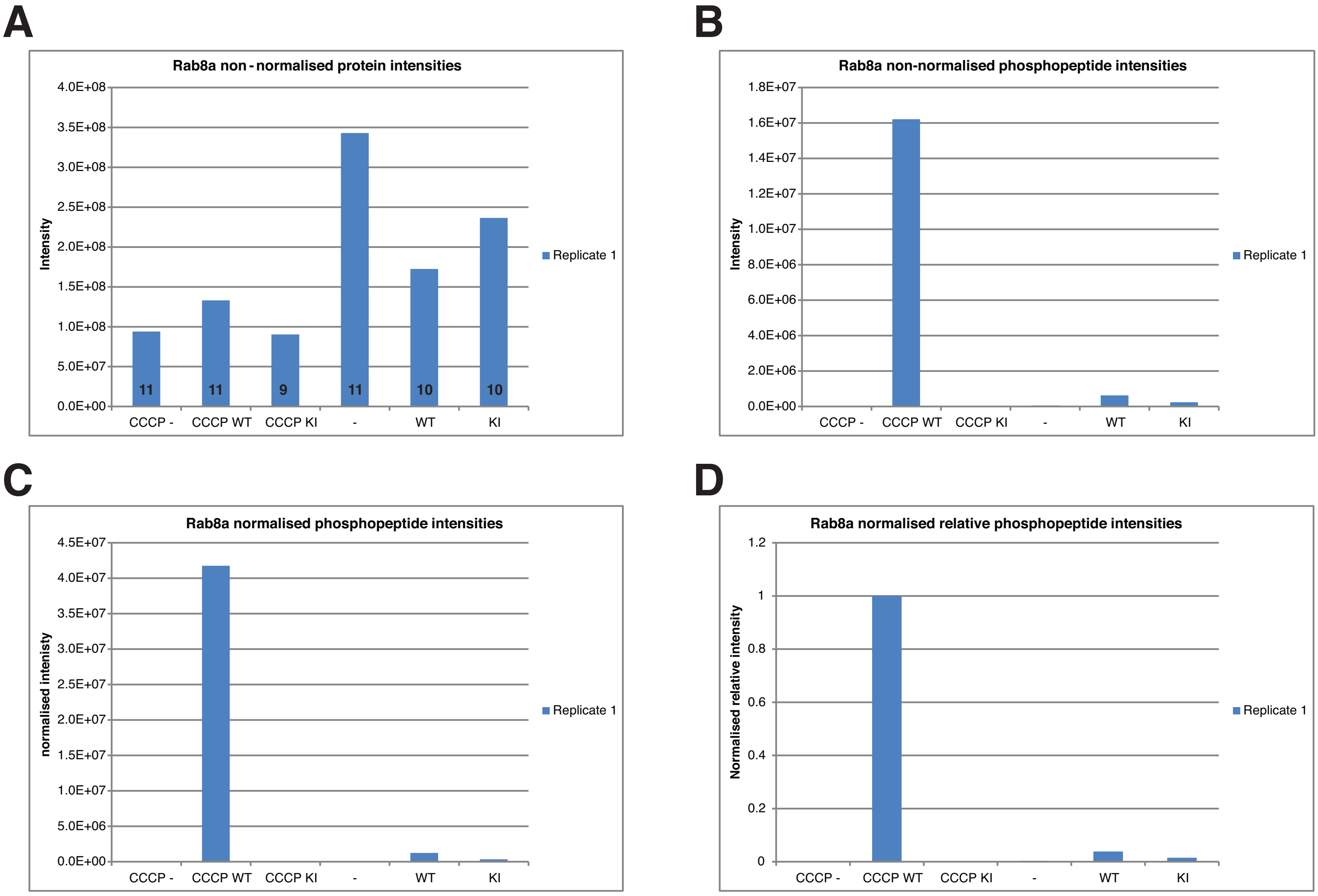

Appendix Fig S5. Rab 8A protein and phosphopeptide intensities from HA-immunoprecipitates. **A:** Non-normalised intensity data for HA-Rab8A from in gel digests of Flp-In TRex HEK293 cells stably transfected with vector controls (-), wild type PINK1 (WT) and kinase inactive PINK1 (KI) either CCCP treated (left side) or non-treated (right side). The number of unique and razor peptides used for quantitation is indicated for each experiment. **B:** Non-normalised intensity data for the phosphopeptide NIEEHApSADVEK around Ser111 of Rab8A from in gel digests of Flp-In TRex HEK293 cells stably transfected with vector controls (-), wild type PINK1 (WT) and kinase inactive PINK1 (KI) either CCCP treated (left side) or non-treated (right side). **C:** As (**B**) but phosphopeptide intensities normalised with protein intensities from (**A**). **D:** Normalised relative phosphopeptide intensities of the same peptide. All intensities were obtained through MaxQuant 1.5.1.7.

Appendix Fig S6

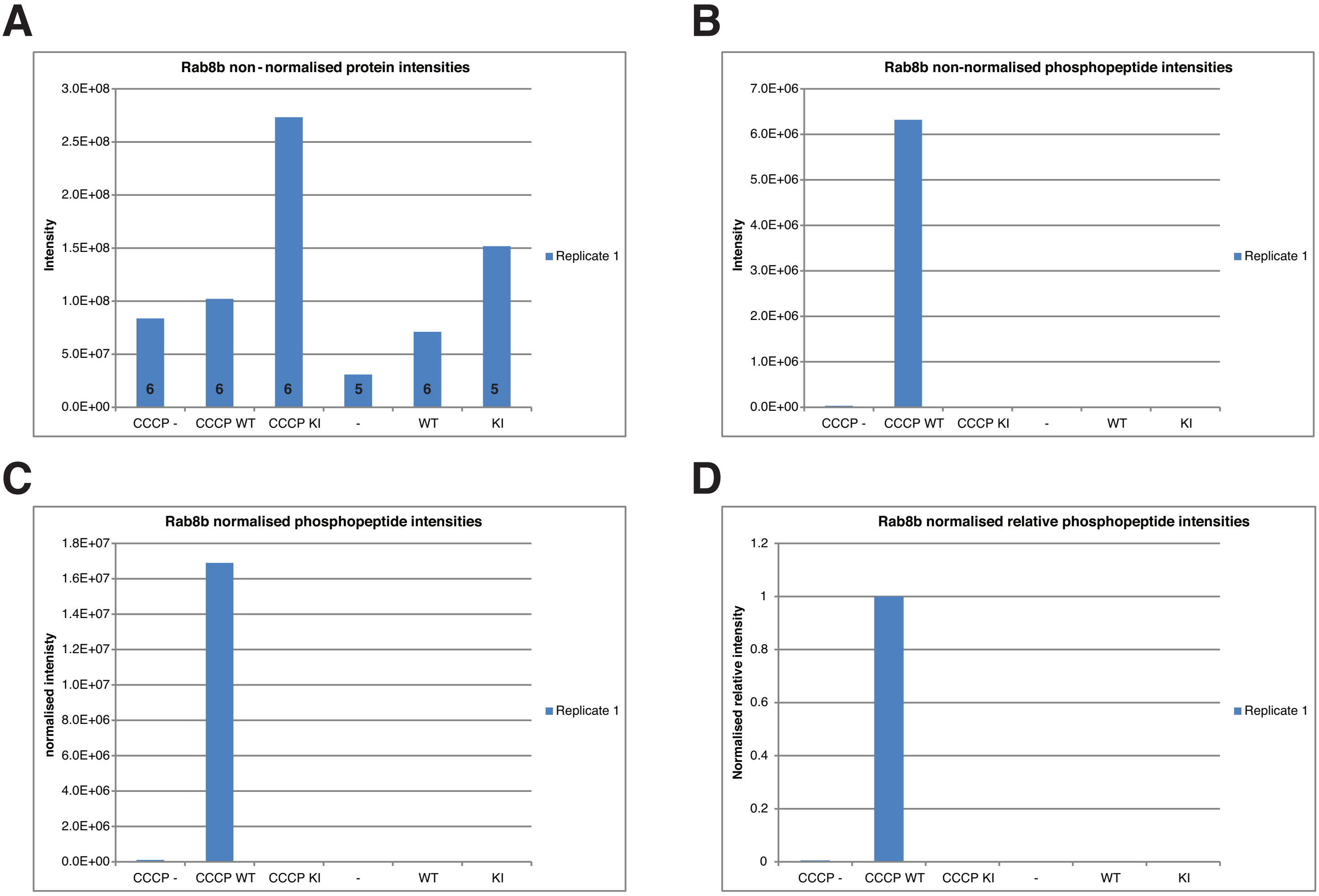

Appendix Fig S6. Rab 8B protein and phosphopeptide intensities from HA-immunoprecipitates.
A: Non-normalised intensity data for HA-Rab8B from in gel digests of Flp-In TRex HEK293 cells stably transfected with vector controls (-), wild type PINK1 (WT) and kinase inactive PINK1 (KI) either CCCP treated (left side) or non-treated (right side). The number of unique and razor peptides used for quantitation is indicated for each experiment. **B:** Non-normalised intensity data for the phosphopeptide NIEEHApSSDVER around Ser111 of Rab8B from in gel digests of Flp-In TRex HEK293 cells stably transfected with vector controls (-), wild type PINK1 (WT) and kinase inactive PINK1 (KI) either CCCP treated (left side) or non-treated (right side). **C:** As (**B**) but phosphopeptide intensities normalised with protein intensities from (**A**). **D:** Normalised relative phosphopeptide intensities of the same peptide. All intensities were obtained through MaxQuant 1.5.1.7.

Appendix Fig S7

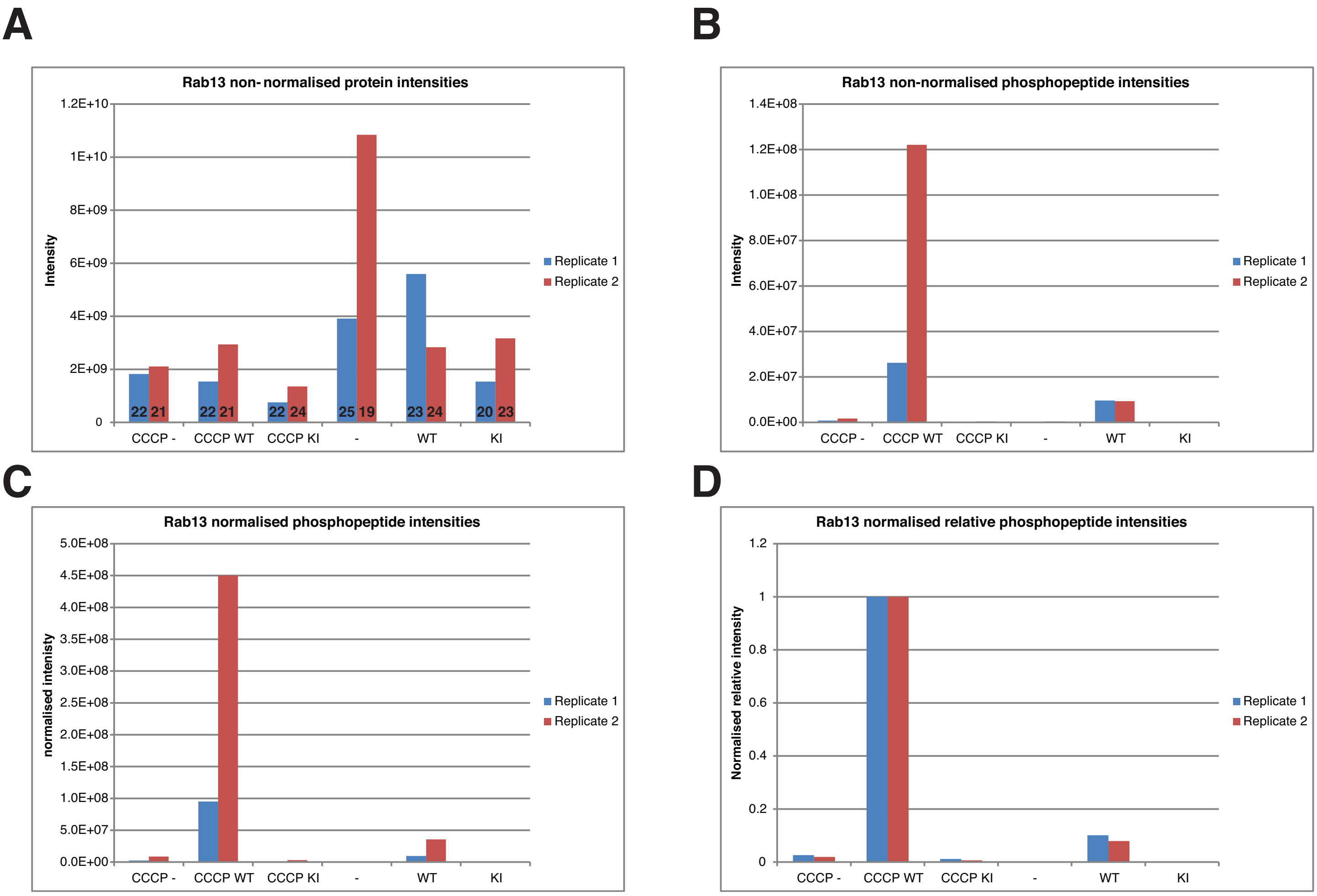

Appendix Fig S7. Rab13 protein and phosphopeptide intensities from HA-immunoprecipitates. **A:** Non-normalised intensity data for HA-Rab13 from in gel digests of Flp-In TRex HEK293 cells stably transfected with vector controls (-), wild type PINK1 (WT) and kinase inactive PINK1 (KI) either CCCP treated (left side) or non-treated (right side). The number of unique and razor peptides used for quantitation is indicated for each experiment. **B:** Non-normalised intensity data for the phosphopeptide SIKENApSAGVER around Ser111 of Rab13 from in gel digests of Flp-In TRex HEK293 cells stably transfected with vector controls (-), wild type PINK1 (WT) and kinase inactive PINK1 (KI) either CCCP treated (left side) or non-treated (right side). The fully tryptically cleaved peptide was not detected. **C:** As (**B**) but phosphopeptide intensities normalised with protein intensities from (**A**). **D:** Normalised relative phosphopeptide intensities of the same peptide. All intensities were obtained through MaxQuant 1.5.1.7.

Appendix Fig S8

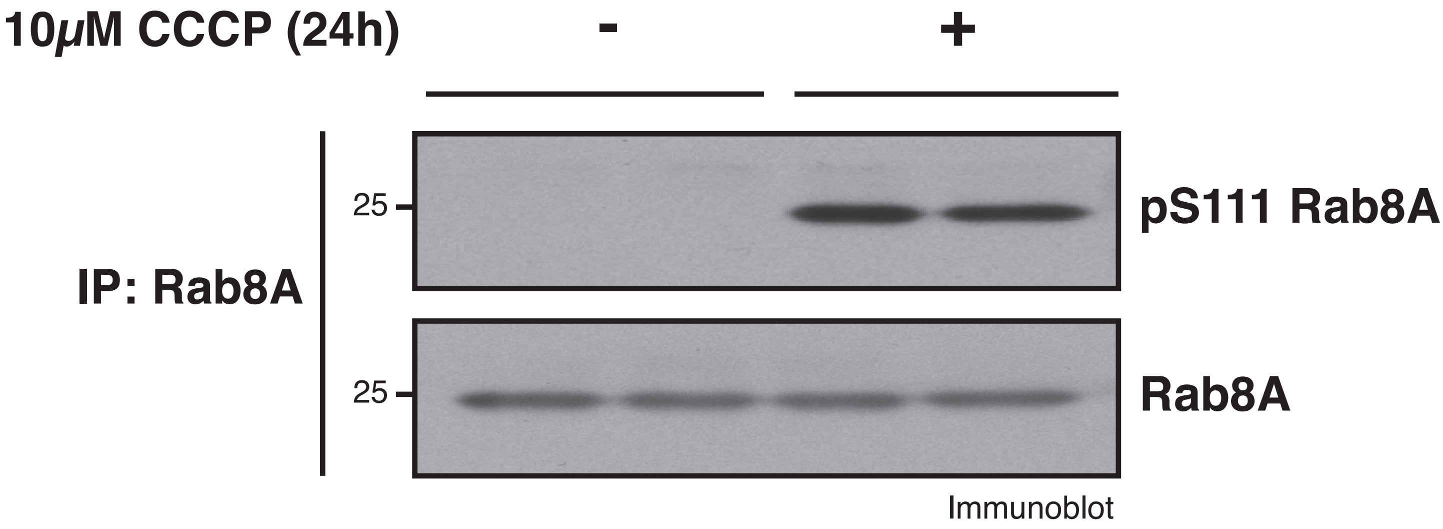

Appendix Fig S8. Endogenous PINK1 regulates endogenous Rab8A Ser^{111} phosphorylation in HEK293 cells. HEK293 cells were treated with DMSO vehicle control or CCCP for 24h. Whole cell lysates (1 mg) were immunoprecipitated with anti-Rab8A (from Cell Signaling Technology) pre-bound with protein A agarose followed by immunoblot with Rab8A phospho-Ser^{111} antibody. Part of immunoprecipitates were used to immunoblot with anti-Rab8A antibody (from Sigma) as loading controls.

Appendix Fig S9

A

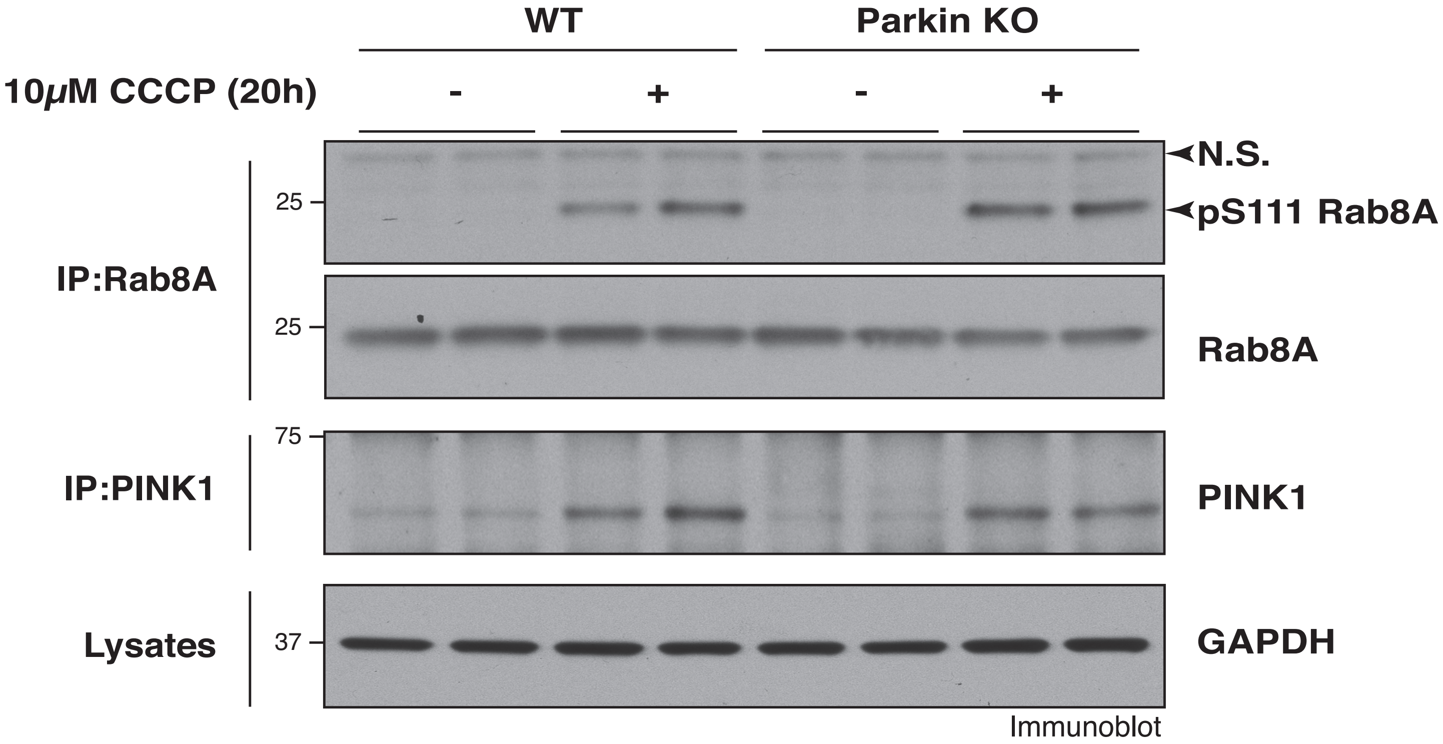

B

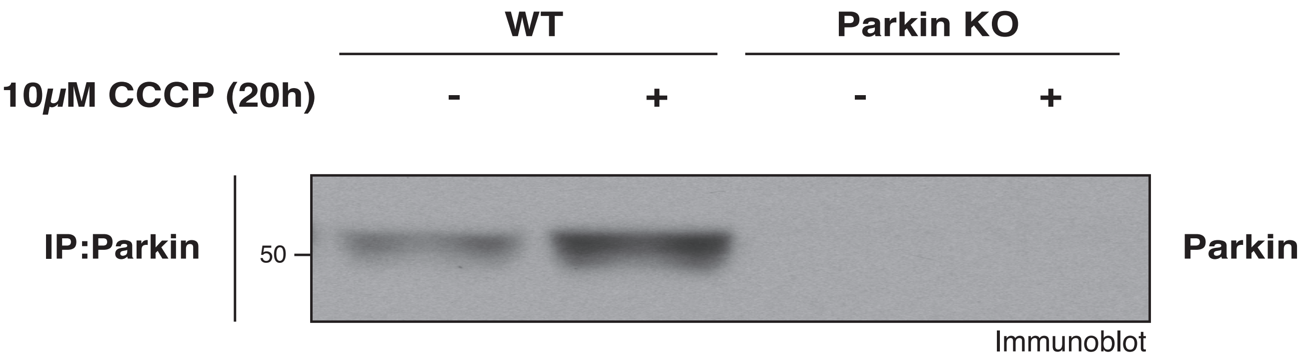

Appendix Fig S9. Rab8A Ser[111] phosphorylation is unaltered in Parkin knockout mouse embryonic fibroblasts (MEFs). MEFs were derived from Parkin knockout embryos or wild-type controls (see methods). Cells were incubated with DMSO or CCCP for 20 h. (A) Whole cell lysates (1 mg) were immunoprecipitated with anti-Rab8A antibody and immunoblotted with total or phospho-Ser[111] Rab8A antibody. Lysates (1 mg) were also subjected to immunoprecipitation with a polyclonal anti-mouse-specific PINK1 antibody and immunoblotted with a different anti-mouse-specific PINK1 antibody. Equal loading of protein extracts was confirmed by GAPDH. (B) Whole cell lysates (20 mg) of Parkin knockout or wild-type MEFs were immunoprecipitated with polyclonal anti-Parkin antibody and immunoblotted with monoclonal Parkin antibody to confirm expression of Parkin in MEFs.

Appendix Fig S10

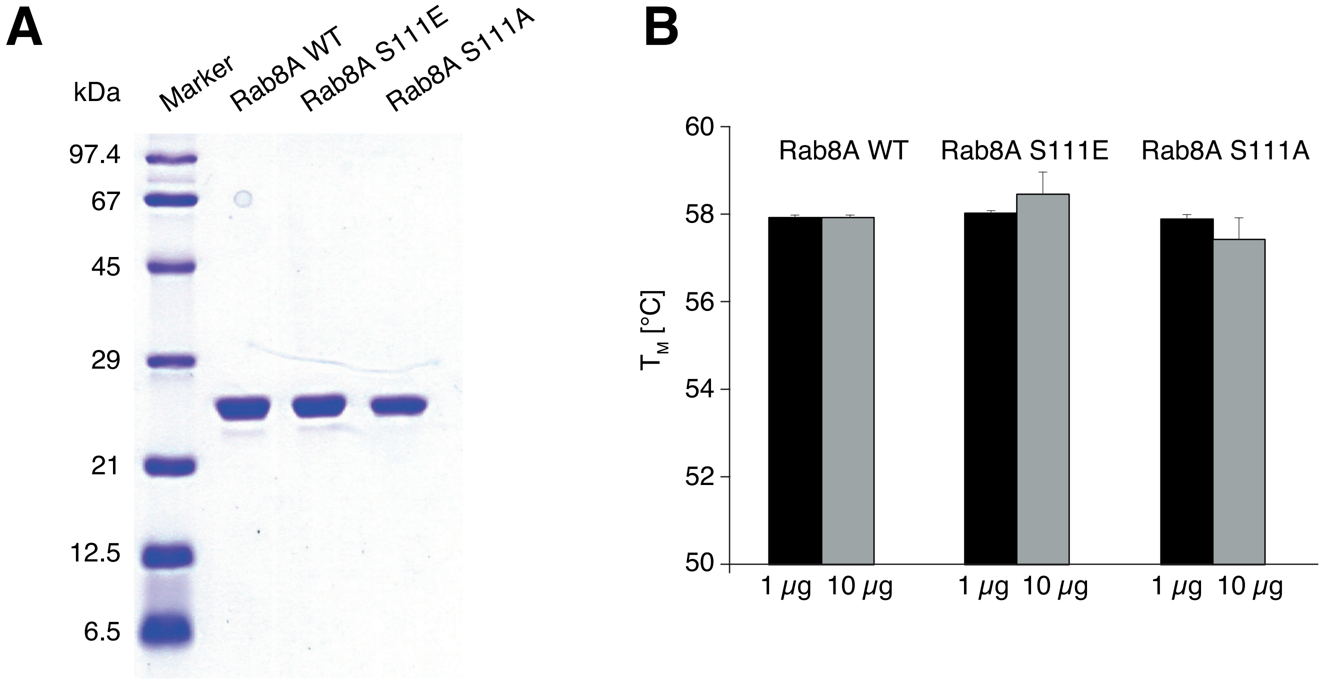

Appendix Fig S10. Purity and stability of recombinant WT and phosphomimetic Rab8A. A: Coomassie stained, 15% SDS-PAGE-gel of purified WT, S111A and S111E Rab8A demonstrating high purity of the prepared proteins. **B**: Stability of Rab8A WT, Rab8A S111A and Rab8A S111E analyzed using a thermal shift assay. The assay was performed in triplicates with 1 and 10 µg of the proteins.

Appendix Fig S11

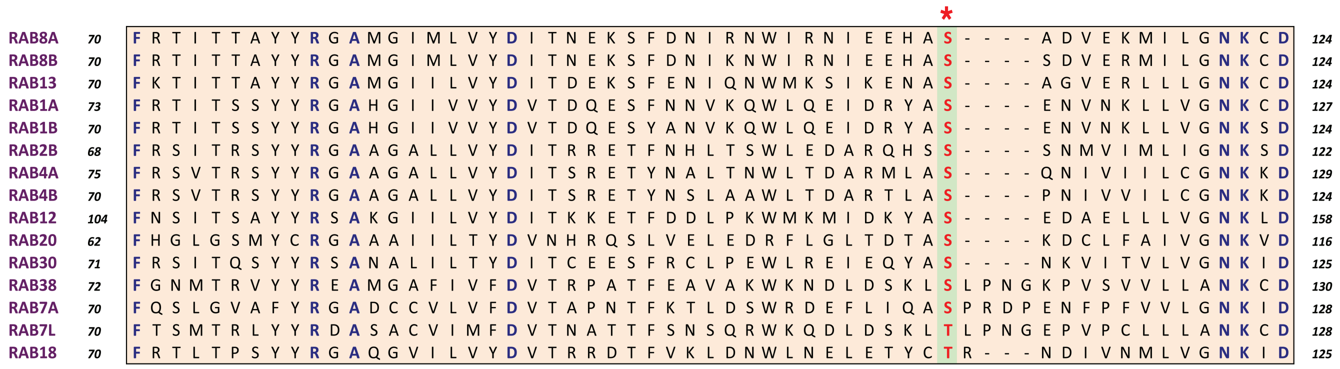

Appendix Fig S11. Ser^{111} of Rab8A is potentially conserved in 15 human Rab GTPases. Multiple sequence alignment of all human Rab GTPases in the region of Ser^{111} of Rab8A reveals 15 Rabs with conservation of phosphorylation site for Ser or Thr. The phosphorylation residue is highlighted with a red asterisk.

Appendix Fig S12

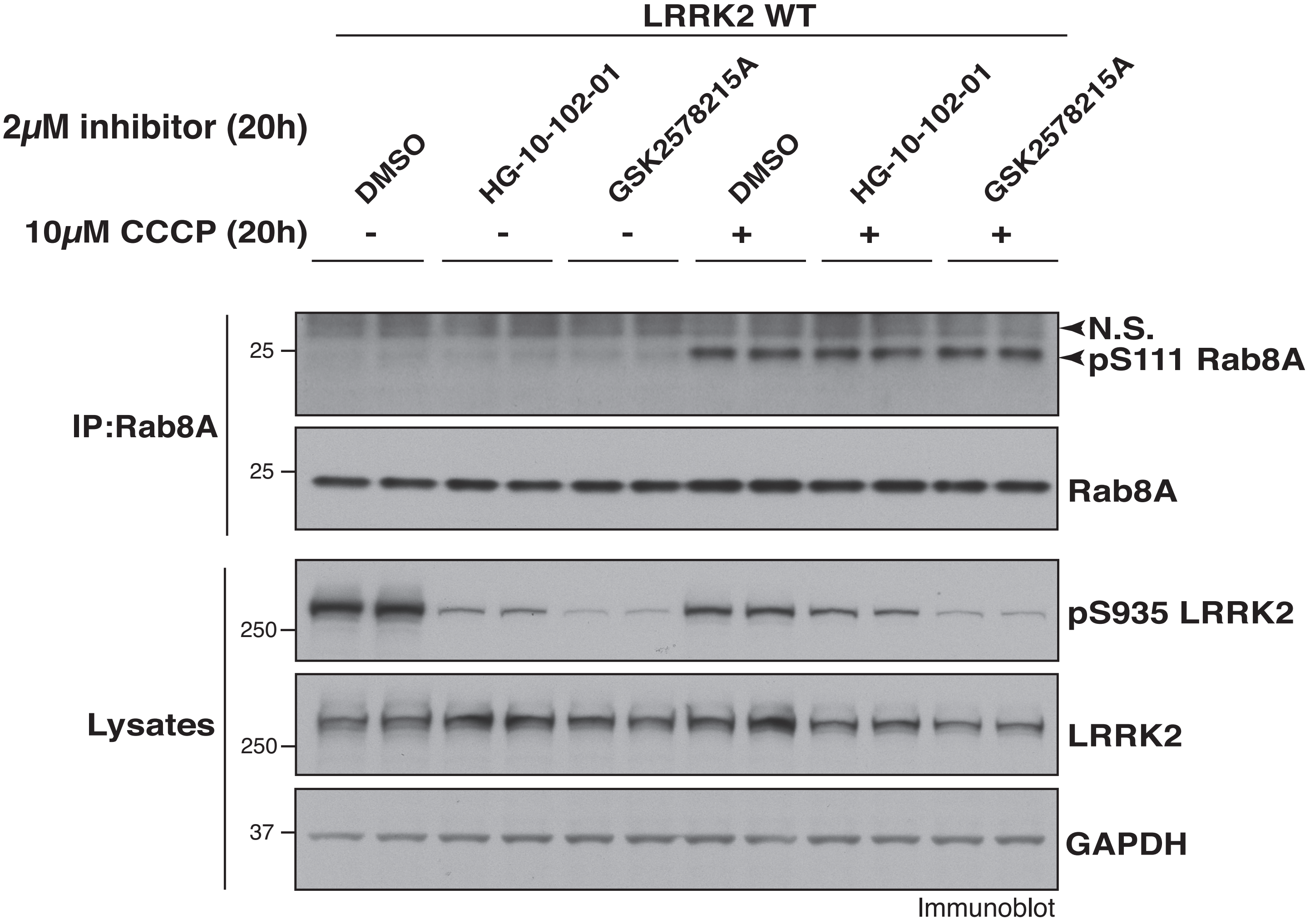

Appendix Fig S12. Inhibition of LRRK2 does not affect CCCP induced Rab8A Ser^{111} phosphoryation. Flp-In TRex HEK293 cells expressing GFP-LRRK2 wild-type (WT) were induced with doxycycline for 24h and treated with DMSO as a vehicle control or one of the two structurally distinct LRRK2 inhibitors in the presence or absence of 10 μM CCCP for 20 h. Whole cell lysates (1 mg) were immunoprecipitated with anti-Rab8A (from Cell Signaling Technology) pre-bound with protein A agarose followed by immunoblot with Rab8A phospho-Ser111 antibody. A fraction of immunoprecipitates was immunoblotted with anti-total Rab8A antibody to confirm equal pulldown. Whole cell lysates (30 μg) were immunoblotted with indicated LRRK2 antibodies to confirm LRRK2 inhibition.
