# Supplementary material for: Phosphoproteomic screening identifies Rab GTPases as novel downstream targets of PINK1
Source: EMBO J. 2015 Oct 16;34(22):2840–61. doi: 10.15252/embj.201591593 (PMC4654935; doi:10.15252/embj.201591593)
Supplement: Supplementary file 2 — Expanded View Figures PDF [file EMBJ-34-2840-s002.pdf]

Expanded View Figures

A

B

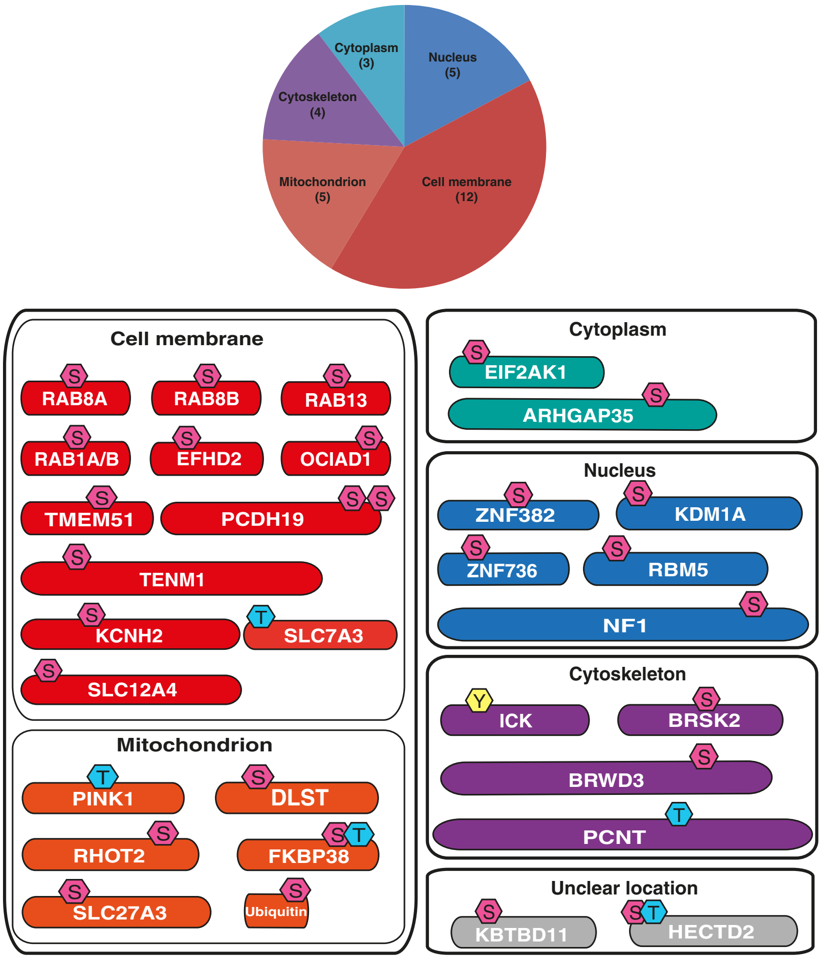

Figure EV1. Analysis of PINK1-dependent phosphoproteins.

A Pie chart analysis showing subcellular localisation of PINK1 up-regulated phosphoproteins. Membrane-bound proteins make up more than half of the regulated phosphoproteins.

B PINK1 up-regulated phosphoproteins sub-grouped according to cell localisation. Magenta hexagon: serine phosphorylation residue; blue hexagon: threonine phosphorylation residue; and yellow hexagon: tyrosine phosphorylation residue.

C Table of PINK1-dependent phosphopeptides that were up-regulated across all four replicates.

C

Uniprot ID	Protein Name	Average Ratio (WT/KI; H/M)	Site	Peptide sequence
P51153	Ras-related protein Rab13	52.2	S111	SIKENA$_{p}$SAGVER
Q92930	Ras-related protein Rab8B	29.9	S111	NIEEHA$_{p}$SSDVER
Q9BXM7	Serine/threonine-protein kinase PINK1	24.3	T257	VALAGEYGAV$_{p}$TYR
P61006	Ras-related protein Rab8A	21.0	S111	NIEEHA$_{p}$SADVEK
P62979	Ubiquitin	16.8	S65	TLSDYNIQKE$_{p}$STLHLVLR
Q14318	Peptidyl-prolyl cis-trans isomerase FKBP38	13.6	S359; T360	$_{p}$S$_{p}$TETALYR
Q96C19	EF-hand domain-containing protein D2	7.35	S74	ADLNQGIGEPQ$_{p}$SPSR

© 2015 The Authors

A

	−6	−5	−4	−3	−2	−1	0	1	2	3	4	5
**Parkin**	C	D	L	D	Q	Q	S	I	V	H	I	V
**Ubiquitin**	Y	N	I	Q	K	E	S	T	L	H	L	V

**Rab8A**	N	I	E	E	H	A	S	A	D	V	E	K
**Rab8B**	N	I	E	E	H	A	S	S	D	V	E	R
**Rab13**	S	I	K	E	N	A	S	A	G	V	E	R
**Rab1A/B**	E	I	D	R	Y	A	S	E	N	V	N	K

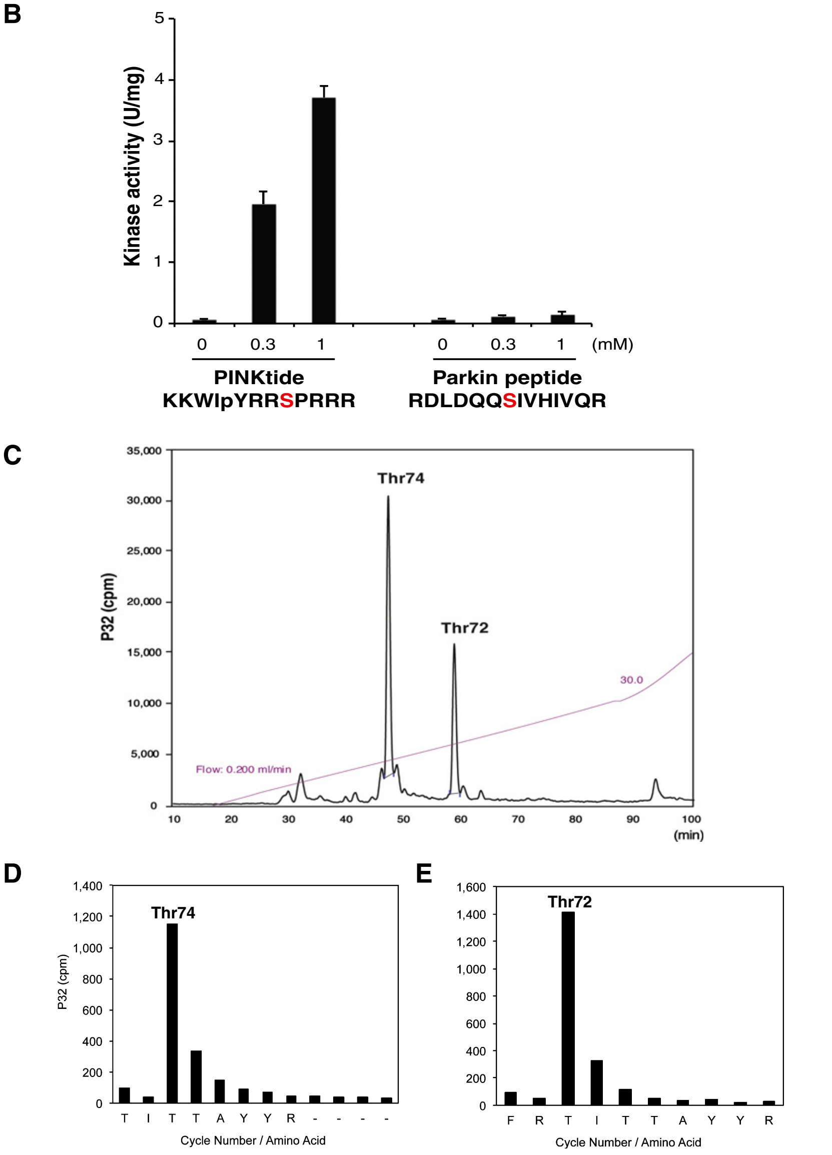

Figure EV2. Evidence that Rab8A Ser[111] is not directly regulated by TcPINK1.

A Sequence alignment of potential PINK1-regulated phosphosites along with Parkin and ubiquitin Ser[65] phosphosite sequence. The residue of phosphosite is kept at 0, and upstream and downstream sequences are marked with negative or positive numbering, respectively.

B Phosphorylation of PINKtide (KKWIpYRR**S**PRRR where S is serine phosphorylated by TcPINK1) and Parkin Ser[65] peptide (RDLDQQ**S**IVHIVQR where S is Ser[65]) by TcPINK1. Full-length MBP-tagged TcPINK1 (1 μg) was incubated in the presence of PINKtide or Parkin peptide at the indicated concentrations and [γ-^{32}P] ATP for 30 min. Reactions were terminated by spotting onto P81 paper and quantified by scintillation counting. Data are means ± SD, $n = 2$.

C Mapping of phosphopeptides on Rab8A after phosphorylation by wild-type TcPINK1. Rab8A (24 μg) was incubated with MBP-fused WT TcPINK1 (50 μg) and Mg^{2+}-[γ-^{32}P] ATP for 2 h. Protein samples were subjected to SDS–PAGE, revealed by colloidal Coomassie blue staining and excised for in-gel trypsin digestion. Peptides were chromatographed on a reversed-phase HPLC Vydac C18 column (218TP5215) equilibrated in 0.1% trifluoroacetic acid and the column developed with a linear acetonitrile gradient fractions (flow rate in 0.2 ml/min; 0.1 ml/fraction) for [γ-^{32}P] Cerenkov counting.

D, E Identification of the Thr72 and Thr74 phosphorylation sites by Edman sequencing and mass spectrometry. Phosphopeptides from peak fractions (C) were sequenced by solid-phase Edman degradation, using a Shimadzu PPSQ-33A sequencer, after the peptides were coupled to Sequelon-arylamine membrane (Applied Biosystems) as described previously (Campbell & Morrice, 2002). The amino acid sequence deduced from the LC-MS-MS is shown using the single-letter code for amino acids.

 © 2015 The Authors

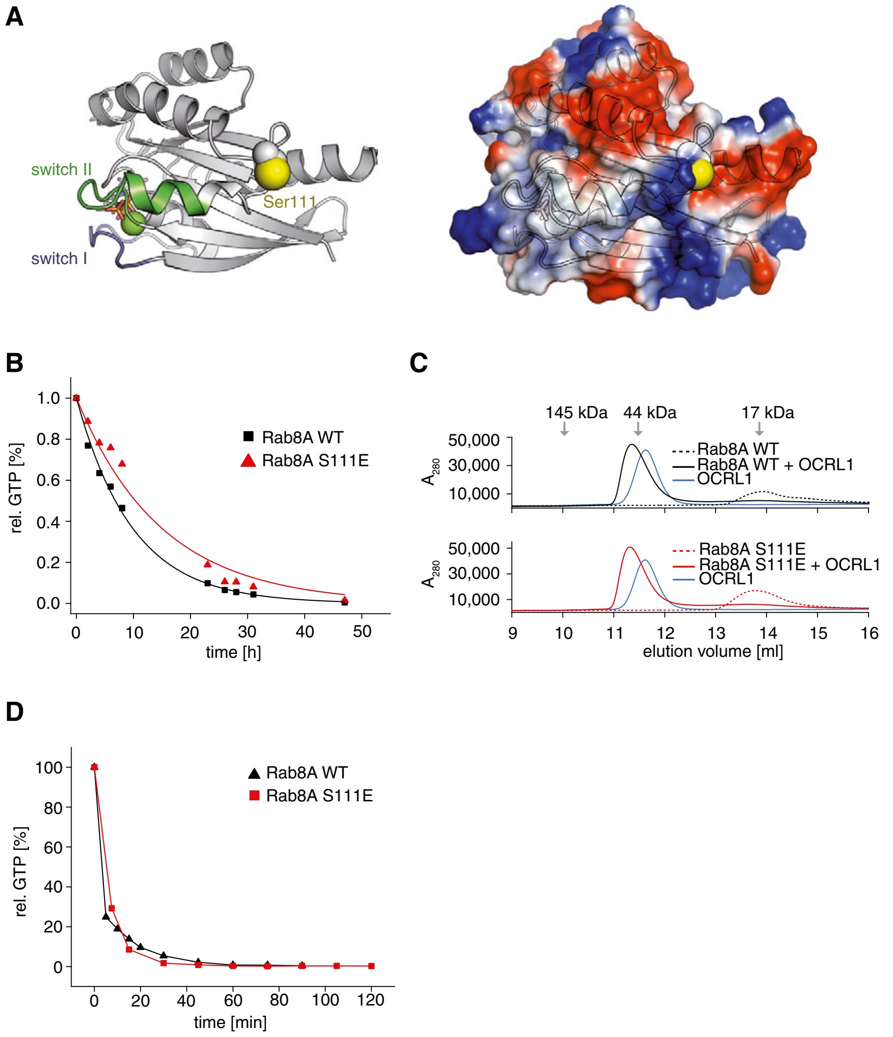

Figure EV3. Functional analysis of recombinant WT and phosphomimetic Rab8A.

A Crystal structure of Rab8A in cartoon (left) and surface (right) representation (Guo *et al*, 2013). The hydroxyl group of residue Ser111 is shown as a yellow sphere and the Mg^{2+}-ion as a green sphere. The GTP analogue is depicted in sticks. The switch regions are coloured blue (switch I) and green (switch II).

B Intrinsic GTPase hydrolysis assay. The rate of intrinsic GTP hydrolysis of Rab8A WT and S111E loaded preparatively with GTP was determined by separating and quantifying GDP and GTP on a reversed-phase column. The relative GTP content has been fitted to a single exponential curve, demonstrating virtually identical rates for Rab8A WT and S111E.

C Analytical gel filtration analysis of OCRL1$_{539\text{-}901}$ interaction with Rab8A WT (upper panel) and Rab8A S111E (lower panel). Standard: γ-globulin (145 kDa) = 10.06 ml, ovalbumin (44 kDa) = 11.51 ml and myoglobulin (17 kDa) = 13.86 ml. OCRL1$_{539\text{–}901}$ (15 μM) and individual Rab proteins (19.5 μM) were each subjected separately and together (after 1 h of incubation) to chromatography.

D GAP-stimulated GTP hydrolysis of the Rab8 phosphomimetic mutant S111E is indistinguishable from Rab8A WT. The TBC1D20$_{1\text{-}305}$ (100 nM)-mediated GTP hydrolysis of Rab8A WT and S111E (30 μM each) preloaded with GTP was monitored by quantitative C18-HPLC in a time-dependent manner.

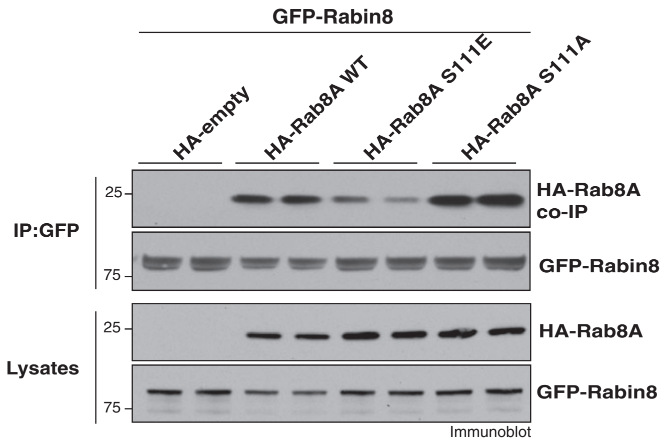

Figure EV4. Decreased S111E Rab8A association with Rabin8 in cells.
Rab8A KO HeLa cells were co-transfected with GFP-Rabin8 and wild-type (WT), S111E or S111A HA-Rab8A. Whole-cell lysates (1 mg) were immunoprecipitated with GFP binder sepharose resin and immunoblotted with anti-HA and anti-GFP antibodies. Lysates were immunoblotted with anti-GFP and anti-HA antibody to confirm equivalent expression of GFP-Rabin8 and HA-Rab8A across all extracts.

© 2015 The Authors

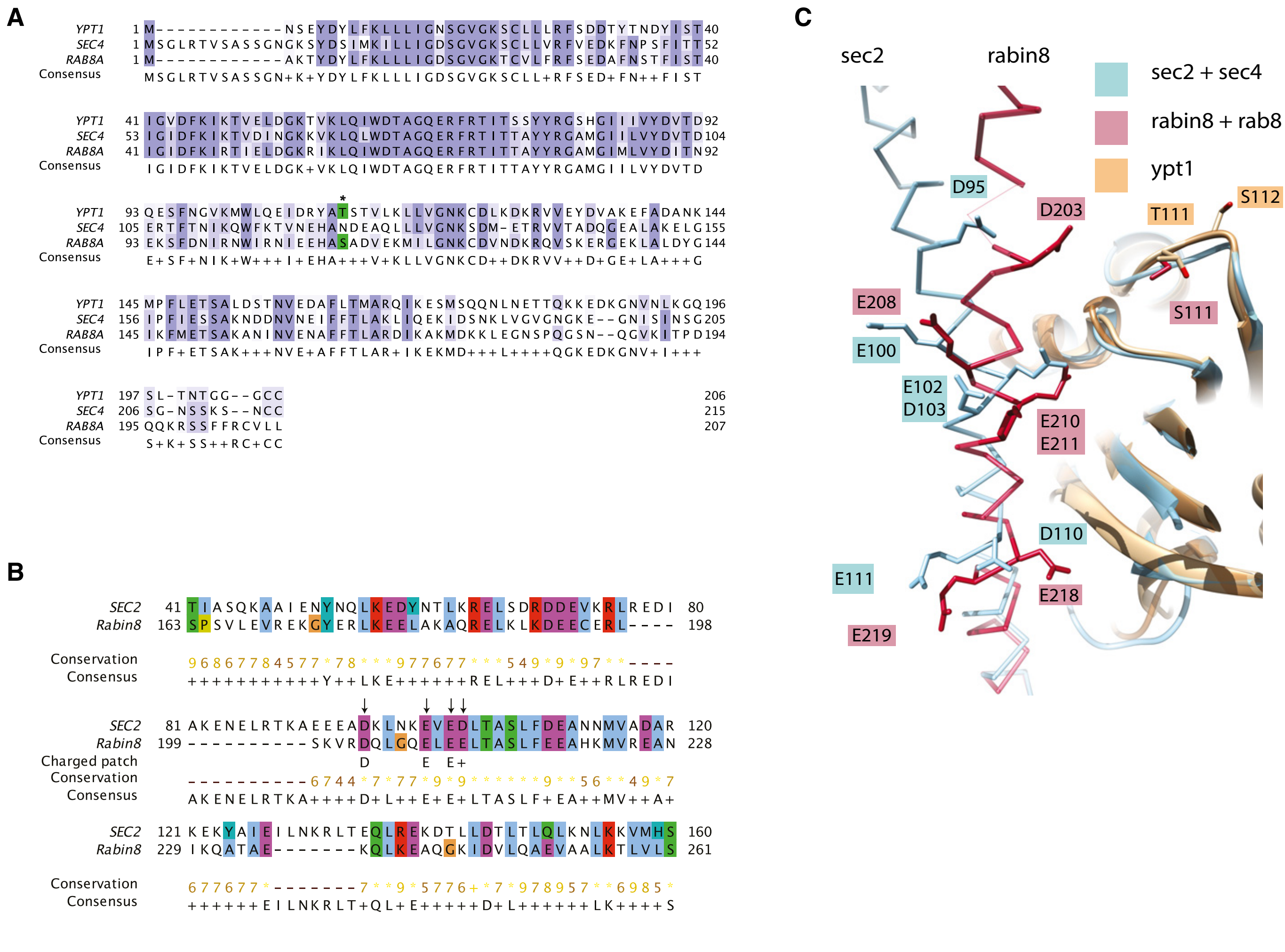

Figure EV5. Comparative analysis of human Rab8 and Rabin8 and yeast orthologues Ypt1/Sec4 and Sec2.

A Multiple sequence alignment of yeast Ypt1, Sec4 and human Rab8A to assess conservation of Rab8A Ser111 (Ser^{111}) phosphosite. Alignment is shaded according to BLOSUM62 similarity, and Ser^{111} site is highlighted in green and marked with an asterisk (*).

B Multiple sequence alignment of yeast Sec2 and human Rabin8. Sequence homology is highlighted by arrows.

C Structural comparison of guanine effector binding region of Sec2 and Rabin8 by superposition of Ypt1, Sec4 and Rab8A (respectively: PDB code 2BCG, yeast crystal structure; PDB code 2OCY, yeast crystal structure; PDB code 4LHY, human crystal structure). Residues highlighted are numbered according to UniProt Q96QFO and P17065 for human Rabin8 and yeast Sec2, respectively. Rabin8 residues D203, E208, E210, E211, E218 and E219 correspond to PDB residue numbering D187, E192, E194, E195, E202 and E203 (PDB code 4LHY; human crystal structure).

 © 2015 The Authors
